# Effects of a work-based critical reflection program for novice nurses

**DOI:** 10.1186/s12909-018-1135-0

**Published:** 2018-02-27

**Authors:** Yeon Hee Kim, Ja Min, Soon Hee Kim, Sujin Shin

**Affiliations:** 1 Asan Medical Center, 88, Olympic-ro 43-Gil, Songpa-gu, Seoul 05505 Republic of Korea; 2 College of Nursing, Ewha Womans University, 52 Ewhayeodae-gil, Seodaemun-gu, Seoul 03760 Republic of Korea

**Keywords:** Critical thinking, Reflection, Communication, Performance

## Abstract

**Background:**

Critical reflection is effective in improving students’ communication abilities and confidence. The aim of this study was to evaluate the effectiveness of a work-based critical reflection program to enhance novice nurses’ clinical critical-thinking abilities, communication competency, and job performance.

**Methods:**

The present study used a quasi-experimental design. From October 2014 to August 2015, we collected data from 44 novice nurses working in an advanced general hospital in S city in Korea. Nurses in the experimental group participated in a critical reflection program for six months. Outcome variables were clinical critical-thinking skills, communication abilities, and job performance. A non-parametric Mann-Whitney U-test and a Wilcoxon rank sum test were selected to evaluate differences in mean ranks and to assess the null hypothesis that the medians were equal across the groups.

**Results:**

The results showed that the clinical critical-thinking skills of those in the experimental group improved significantly (*p* = 0.003). The differences in mean ranks of communication ability between two groups was significantly statistically different (*p* = 0.028). Job performance improved significantly in both the experimental group and the control group, so there was no statistical difference (*p* = 0.294).

**Conclusions:**

We therefore suggest that a critical reflection program be considered an essential tool for improving critical thinking and communication abilities among novice nurses who need to adapt to the clinical environment as quickly as possible. Further, we suggest conducting research into critical reflection programs among larger and more diverse samples.

## Background

As nurses are significant decision-makers within healthcare systems, their clinical judgments and decisions are required to contribute to the quality of such systems [1]. Nursing education essentially aims to advance the practical application of theoretical knowledge [2], so it is important to provide learners with opportunities for experiential learning. In this respect, critical reflection enables learners to develop self-awareness and doubt, allowing them to gain a comprehensive perspective regarding particular issues [3]. In other words, critical reflection is a fundamental component of clinical nursing practice, potentially impacting personal and professional development.

Nursing students and nurses are required to solve problems in complex clinical situations [4]. In this context, continual critical reflection can bridge the gap between theory and practice and stimulate personal and professional development [5]. Therefore, training in critical thinking and clinical decision-making ought to form a vital part of not only university education but also professional development in clinical fields.

Critical reflection is a meta-cognitive skill and a key mechanism in the process of critical thinking [6]. Furthermore, critical reflection is part of both experiential learning and transformative learning, and critical reflection acquired from practice enhances learning [7]. According to the model proposed by Cox [8], clinical education consists of two main cycles, namely the experience cycle and the explanation cycle. Critical reflection forms the core of Cox’s model.

In light of the above, it appears imperative for educators to develop learners’ critical reflection ability. Critical reflection is a valuable learning process for enhancing nurse-patient communication [9]. Critical reflection in analyzing the outcomes of nursing practice has been found to be effective in improving learners’ communication competencies [10], and it has been reported that such improvement in communication abilities has a positive effect on nursing job performance [11]. With the increased emphasis on nurses’ judgment skills, nursing students ought to receive work-related education by which to develop their clinical critical-thinking skills on the basis of actual nursing contexts. Additionally, one qualitative study reported that reflective practice in professional development reduced stress and improved patient care capabilities [12].

However, there has been little research on the efficacy of work-based educational intervention concerning critical reflection training and there has been little empirical evidence of the effectiveness of critical reflection.

Against this background, the present research aimed to quantify the effects of critical reflection training among novice nurses and offer strategic guidelines on how best to advance the effects of critical reflection training. Specifically, the purpose of this study was to evaluate the effectiveness of a particular work-based critical reflection program in enhancing novice nurses’ clinical critical-thinking abilities, communication competency, and job performance.

## Methods

### Research design

This study employed a quasi-experimental research design carried out to identify the effect of a work-based critical reflection program. We received ethical approval for the study from the Institute of Review Board of Asan Medical Center (2014–1021). Following IRB approval, we implemented a three phase program: 1) development of the work-based Critical Reflection Program, 2) training of eight nurses to facilitate the program as Reflective Practitioners (RPs), and 3) implementation of the Critical Reflection Training Program and measurement of the effects on critical thinking, communication, and performance.

### Samples and participants

The participants of this study were novice nurses stationed at an advanced general hospital. Novice nurses were eligible to participate if they met the following criteria: they were a (1) new graduate nurse (2) who had completed their orientation of approximately two months, and who (3) had not experienced any other critical reflection program. The exclusion criterion was unwillingness to participate.

The required sample size of at least 44 participants was determined using the G*power 3.1 program for the matched paired t-test with a significance level of 0.05, power of 0.9, and effect size of 0.5. The effect size was based on findings that analyzed the effects of critical reflection research training on the clinical decision making of new graduate nurses [13], because no research has verified this using the same variables as in this study. To allow for dropout, 50 potential participants were initially selected and randomly assigned to either the experimental or control group. Of the 25 participants recruited for the experimental group, one dropped out, leaving 24 experimental participants. Of the 24 participants recruited for the control group, four dropped out, leaving 20 control participants.

### Training procedure for the reflective practitioners

For the purposes of this study, authors developed a Critical Reflection (CR) Training Program on the basis of Kolb’s [14] learning cycle model and Cox’s [8] model for clinical teaching. The developed program was reviewed by two educational experts. We explained the purpose of the program to potential reflective practitioners (RPs), and then we recruited eight nurses who voluntarily agreed to be trained as RPs. The RPs were trained by means of a four-week/two hours per week program. We provided RPs with the manual for the critical reflection program. One of the researchers conducted the RP training which consisted of: 1) experiential learning and critical reflection, 2) reinforcement of clinical competence through critical reflection, 3) application of critical reflection in nursing fields, and 4) strategies for guiding critical reflection. To maintain fidelity of the intervention and maintain reliability among RPs, time was alloted for RPs to share their experiences and discuss specific difficulties.

### Work-based critical reflection program for novice nurses

We explained the purpose of the program, and novice nurses voluntarily agreed to participate. Each novice nurse in the experimental group was partnered with a trained RP who had worked in similar area. After setting a mutual goal, each experimental participant underwent six months of critical introspection training.

The training program for critical reflection consisted of a number of phases. The opening phase offered an overview, identified needs, introduced the detailed procedure of critical reflection, and established a learning agreement. Following this, in the beginning stage, the participants wrote critical reflection journals on the basis of a case study developed for learning, upon which the RPs provided feedback. In the practice phase, the participants selected actual clinical cases, wrote critical reflection journals, and shared their experience and RP’s feedback. The form of the critical reflection journal, and the basic questions with which the participants were presented were based on the essential elements of debriefing proposed by Zigmont, Kappus, and Sudikoff [15], including relaxation, discovery, and deepening. The questions included the following: “What were the circumstances of the case? Was there prioritization of patient data? What did you do well as the nurse in charge? What did you consider important in this situation? What do you think needs to be improved, and what was done incorrectly? How might you apply what you learned in further similar contexts?” These questions helped the participants to make connections between the experience and explanation cycles.

### Measurement

Data on participants’ general characteristics, clinical critical-thinking skills, communication competency, and job performance were collected in both the experimental and control groups. All participants were asked to complete (a) a questionnaire covering demographic information; (b) the Clinical Critical Thinking Skill test (CCTS) [16]; (c) the Global Interpersonal Communication Competency Scale (GICCS) [17]; and (d) the performance measurement scale [18].

The CCTS proved the reliability and validity of Shin et al. [16], which contained 19 questions; a correct answer scores one point and an incorrect answer zero, yielding a total score out of 19, with a higher score implying better clinical critical-thinking skills. The reliability of the instrument was measured by means of Cronbach’s α, which was 0.69 at pre-testing and 0.56 at post-testing.

The GICCS was developed and validated by Hur [17], and consists of 15 five-point Likert scale items. A higher score indicates better communicative competence. At the time when the tool was developed, Cronbach’s α was 0.72, and 0.76 at pre-testing and 0.70 at post-testing.

The job performance scale was developed by Ko et al. [18]. It consists of 17 five-point Likert scale items, and a higher score indicates better job performance. At the time of development, Cronbach’s α was 0.92, and 0.92 at pre-testing and 0.94 at post-testing.

### Statistical analysis

Data were analyzed using descriptive and analytic statistics using IBM SPSS 22.0. Chi-squared test was used to compare the results of proportions. The Kolmogorov-Smirnov test result was less than 0.05 with a probability of 0.039. The null hypothesis was rejected and the nonparametric test was used. Based on the normality test results, the non-parametric Mann-Whitney U-test and Wilcoxon rank sum test was selected to evaluate differences in mean ranks and to assess the null hypothesis that the medians were equal across groups. The statistical significance level was set at *p* < 0.05.

## Results

The data from the demographic questionnaire were used to ensure homogeneity between the experimental and control groups in terms of their general characteristics. No statistically significant differences were found, indicating that the two groups were largely homogeneous (Table 1). The mean age of the control group was 23.2 (± 1.16) years, and the mean age of the experimental group was 23.3 (± 1.45) years. In terms of gender, 90.0% and 91.7% of participants in the control group and the experimental group were women, respectively. A total of 75.0% of the control group and 79.2% of the experimental group answered that they had completed the critical thinking course. A total of 65.0% of the control group and 66.7% of the experimental group had experience in problem-based learning.

**Table 1:** Homogeneity between experimental group and control group

	Control group (*n* = 20)	Experimental group (*n* = 24)	*p*
Age (year)	23.2 (1.16)	23.3 (1.45)	0.363^b^
Gender			
Male	2 (10.0%)	2 (8.3%)	1.000^a^
Female	18 (90.0%)	22 (91.7%)	
CT course			
Taken	15 (75.0%)	19 (79.2%)	1.000^a^
Not taken	5 (25.0%)	5 (20.8%)	
PBL			
Taken	13 (65.0%)	16 (66.7%)	1.000^a^
Not taken	7 (35.0%)	8 (33.3%)	
CCTS	14.0 (3.50)^c^	14.0 (1.00)^c^	0.482^b^
Communication	53.0 (4.75)^c^	57.5 (10.55)^c^	0.143^b^
Job performance	52.0 (11.00)^c^	53.0 (15.00)^c^	0.724^b^

*CT* Critical thinking, *PBL* Problem-based learning, *CCTS* Clinical critical thinking skills

^a^Chi- square test

^b^Mann-Whitney U test

^c^Median (Interquartile range)

In terms of the efficacy of the work-based critical reflection program, the clinical critical-thinking skills improved significantly in the experimental group (*p* = 0.003). The differences in mean rank of communication ability between the two groups were significantly different (*p* = 0.028). Job performance improved significantly in both the experimental group and the control group, so there were no statistical differences (*p* = 0.294) (Table 2).

**Table 2:** Comparison of mean and standard deviation of dependent variables in the experimental and control groups before and after intervention

	Control group		Intervention group		*p*
	Median	IQR	Median	IQR	
CCTS					
Before	14.0	3.50	14.0	1.00	0.482^a^
After	13.0	3.00	16.0	2.50	
Difference	1.0	0.50	−2.0	−1.50	0.003^b^
Communication					
Before	53.0	7.75	57.5	10.55	0.143^a^
After	53.5	4.75	53.5	8.00	
Difference	−0.5	2.00	4.0	2.55	0.028^b^
Job performance					
Before	53.0	15.00	52.0	11.00	0.724^a^
After	59.0	13.00	59.5	13.75	
Difference	−6.0	1.25	−7.5	−2.75	0.294^b^

*IQR* Interquartile range, *CCTS* Clinical critical thinking skills

^a^Mann-Whitney U test

^b^Wilcoxon rank sum test

## Discussion

The main findings of this study show that the work-based critical reflection program had a positive effect on participants’ clinical critical-thinking abilities (Table 2), in line with previous research findings that critical thinking skills improved through critical thinking training in a fellowship program for nurses [19]. The CCTS scores before intervention are similar to those of studies using the same scales for nursing students [16], but in the score after intervention is higher. In this study, Cronbach’s α for CCTS was 0.56~ 0.69. This value indicates a relatively low internal reliability value. This is because the CCTS elicited a response of 0 or 1, similar to dichotomous variables. However, each item on the CCTS had the correct answer, and it was suggested that the difficulty and discrimination at the time of development was good.

Cox [8] proposes that learning may be improved when the experience cycle moves on to the explanation cycle, and the learning cycle may lead to working knowledge when students go through reflection and explanation stages, respectively. Adequate preparation for a following patient is only possible when students have gone through this explanation cycle. At this point, teachers should avoid providing students with a detailed explanation about whether or not their decisions are correct, and rather offer an atmosphere in which they can develop their own hypothesis and go through the process of verifying it. This approach may help enhance clinical reasoning and critical reflection [20]. The RPs who participated in this study learned to train novice nurses to ask relevant questions and arrive at reasonable answers. Rather than offering direct solutions, they provided participants with direction in finding appropriate answers to their questions. Such questions allowed novice nurse learners to move from the experience cycle to the explanation cycle on their own.

However, the training program did not improve communication ability. This finding was not consistent with those of Farrington and Townsend [9]. In the experimental groups, that the results of post-scoring were lower than the pre-scored results is very interesting, and it is difficult to compare it because there is little research that measures, using the same tool, change in the communication ability score of new nurses. The preliminary survey responses are the results of pre-exposure to the critical reflection program, showing that new nurses expected to enter the clinic after graduation and that they had positive expectations of their communication ability. However, the intervention test was conducted after newly graduated nurses had provided nursing care to patients at the end of the training period, during which time they encountered complex and difficult communication situations in the clinical field.

Finally, with regard to job performance, both experimental and control groups improved significantly from before to after the intervention. This suggests that job performance is likely to improve over time, irrespective of whether or not critical thinking training is offered. Such improvement may indeed have been expected in the present context, as the hospital in which the study was performed had adopted a two-month preceptorship program for novice nurses. Thus, the improvement of job performance in the control group implies the effectiveness of hands-on training through such preceptorship.

Nevertheless, in contrast to the control group, which only received the preceptorship, the experimental group improved in terms of not only job performance but also clinical critical-thinking skills and communication competency.

Recently, in Korea, it has been noted that the turnover rate for nurses with less than one year of practical experience is twice as high as that for other nurses, at 33.6% [21]. According to previous qualitative studies, novice nurses suffer a range of negative experiences: “being emotionally hurt by senior nurses … fear of being scorned at … being scared of senior nurses … having low confidence” [22], and “experiencing a gap between school education and practice” [23]. Because of the special nature of hospital work, hospitals have a complex organizational structure with varying types of relationships among professionals. The role of the nurse unit, or ward manager, is pivotal in influencing the learning environment for new nurses, and there is a clear association between positive nursing role-models and a supportive learning environment [24].

Also, the results of this study support the claim that work-based educational interventions are effective for strengthening nurses’ resilience through critical reflection [25]. Critical reflection provided a positive way to approach practice, so inclusion of education on reflection should be considered an essential component of novice orientation and preceptor training programs [26]. Therefore, it is important for health professionals to possess clinical decision-making and communication abilities. Novice nurses are not exempt from this requirement, so to prevent them from leaving their current posts, it is critical to help them improve their communication abilities and confidence, and to provide them with not only elementary training for nursing but also experiential learning that can close the gap between theoretical knowledge and practice.

However, the present study had certain limitations. Firstly, as the participants were drawn from a single hospital in South Korea, the extent to which the findings may be generalized is limited. Secondly, the sample size was limited to that required for statistical power. Thirdly, in terms of fidelity of intervention, although we developed and provided the manual to RPs and monitored them twice during the intervention, we could not control the fidelity depending on the characteristics of each RPs. Further research with critical thinking training programs among more diverse and larger samples may lend further support to the present findings.

## Conclusions

The work-based critical reflection program not only had a positive effect on clinical decisions through communication and clinical critical-thinking ability but also helped novice nurses to adapt to their working environment with ease. For these reasons, a critical reflection program could be considered an essential tool for improving critical thinking among novice nurses who need to adapt to the clinical environment as promptly as possible. However, our study had some limitations, as mentioned above; therefore we recommend that further studies include larger and more diverse samples.

## Abbreviations

CCTS: Clinical Critical Thinking Skill
CR: Critical reflection
CT: Critical thinking
PBL: Problem-based learning
RP: Reflective practitioner
